# Effect of the Notch1-mediated PI3K-Akt-mTOR pathway in human osteosarcoma

**DOI:** 10.18632/aging.203261

**Published:** 2021-09-08

**Authors:** Kexiang Zhang, Song Wu, Hongwei Wu, Li Liu, Jiahui Zhou

**Affiliations:** 1 Department of Orthopedics, Third Xiangya Hospital, Central South University, Changsha 410013, Hunan Province, China; 2 Department of Orthopedics, Hunan Cancer Hospital and The Affiliated Cancer Hospital of Xiangya School of Medicine, Central South University, Changsha 410013, Hunan Province, China

**Keywords:** osteosarcoma, Notch1, autophagy, PI3K/Akt/mTOR

## Abstract

Purpose: Osteosarcoma is one of the most common malignant bone tumours in early adolescence. The incidence rate of osteosarcoma has stagnated over the past 30 years, highlighting the need to develop novel therapies. In osteosarcoma cells, Notch1 expression is absent, and the Notch1 pathway is related to cancer cell proliferation, apoptosis and autophagy. Our study aimed to investigate the role of Notch1 in osteosarcoma development.

Methods: We measured NICD1 expression induced by doxycycline treatment at various concentrations. The viability of human osteosarcoma cells (MG-63) induced by doxycycline was measured. Flow cytometry and cell apoptosis analysis were conducted to measure the effect of Notch1 on the cell cycle of human osteosarcoma cells. We also used a GFP-LC3 plasmid to detect Notch1-induced autophagy in MG-63 cells. Western blotting was conducted to analyse expression of the PI3K/Akt/mTOR signalling pathway through Notch1 induction by doxycycline.

Results: In this study, we demonstrated that Notch1 activation by doxycycline potently suppressed cell proliferation by inducing S phase arrest in osteosarcoma cells. Doxycycline-induced Notch1 activation also induced apoptosis and autophagy in osteosarcoma cells. Moreover, we found that Notch1 inhibited PI3K/Akt/mTOR signalling to induce apoptosis and autophagy.

Conclusion: In summary, our results revealed that Notch1 activation by doxycycline induces S phase arrest, apoptosis and autophagy by blocking PI3K/Akt/mTOR signalling in human osteosarcoma cells. Notch1 may be a potential clinical antitumour target for osteosarcoma therapy.

## INTRODUCTION

Osteosarcoma (OS) is the most common primary bone malignancy in children and adolescents [1]. Half of osteosarcoma patients have pulmonary metastases due to early metastases. Because of its life-threatening nature, the relatively low incidence does not fully reflect the real burden of OS on patients and communities [2]. Preoperative inductive and postoperative immunogenic chemotherapy have significantly increased the 5-year survival rate from 20% to 70% [3]. However, the survival rate has remained almost unchanged for three decades since the introduction of multi-drug chemotherapy [4].

Autophagy is the primary means by which eukaryotes have evolved to degrade autologous organelles or proteins [5], regulating and controlling cell proliferation and function. Increasing studies have shown that autophagy plays an important role in the development of malignant tumours [6]. Microtubule-associated protein 1 light chain 3 (LC3), which is located at the membrane surface of the pre-autophagosome and autophagosome, is usually used as a specific marker of autophagy [7]. Beclin-1 is also regarded as a marker of autophagy and inhibits tumour growth through the enhancement of autophagy [8]. In addition, the phosphatidylinositol 3-kinase/Akt/mTOR signalling pathway plays an important role in tumour cell proliferation, apoptosis, autophagy, relapse and metastasis, as well as the degradation of the extracellular matrix [9]. Hence, drugs inhibiting PI3K/Akt/mTOR are increasingly valued in tumour research.

The Notch family (Notch1-4) of heterodimeric transmembrane receptors regulates cell differentiation, proliferation, and apoptosis and plays a critical role in development [10]. Increasing evidence demonstrates that Notch1 regulates PI3K-Akt-mTOR signalling in tumours, although many details are still emerging [11]. In MDA-MB-231 cells, in which Notch1 is constitutively activated, p-Akt is upregulated, indicating activation of the PI3K/Akt pathway [12]. The mammalian target of rapamycin complex (mTOR) pathway is a major point of convergence of Notch1 and PI3K-Akt signalling and promotes the growth of T-ALL cells [13–15].

There is still controversy regarding the role of autophagy in tumour treatment [16], and the inhibitory and protective effects of autophagy during tumour treatment need to be further elaborated. In the present study, human osteosarcoma cell lines were treated with a Notch1 inducer, and changes in cell proliferation, the cell cycle and autophagy were analysed. The aim was to investigate changes in autophagic activity and the molecular mechanism of Notch1-mediated PI3K/Akt/mTOR signalling during autophagy to provide a theoretical basis for the treatment of osteosarcoma.

## RESULTS

### Notch1 inhibits osteosarcoma cell viability

**Figure 1:**
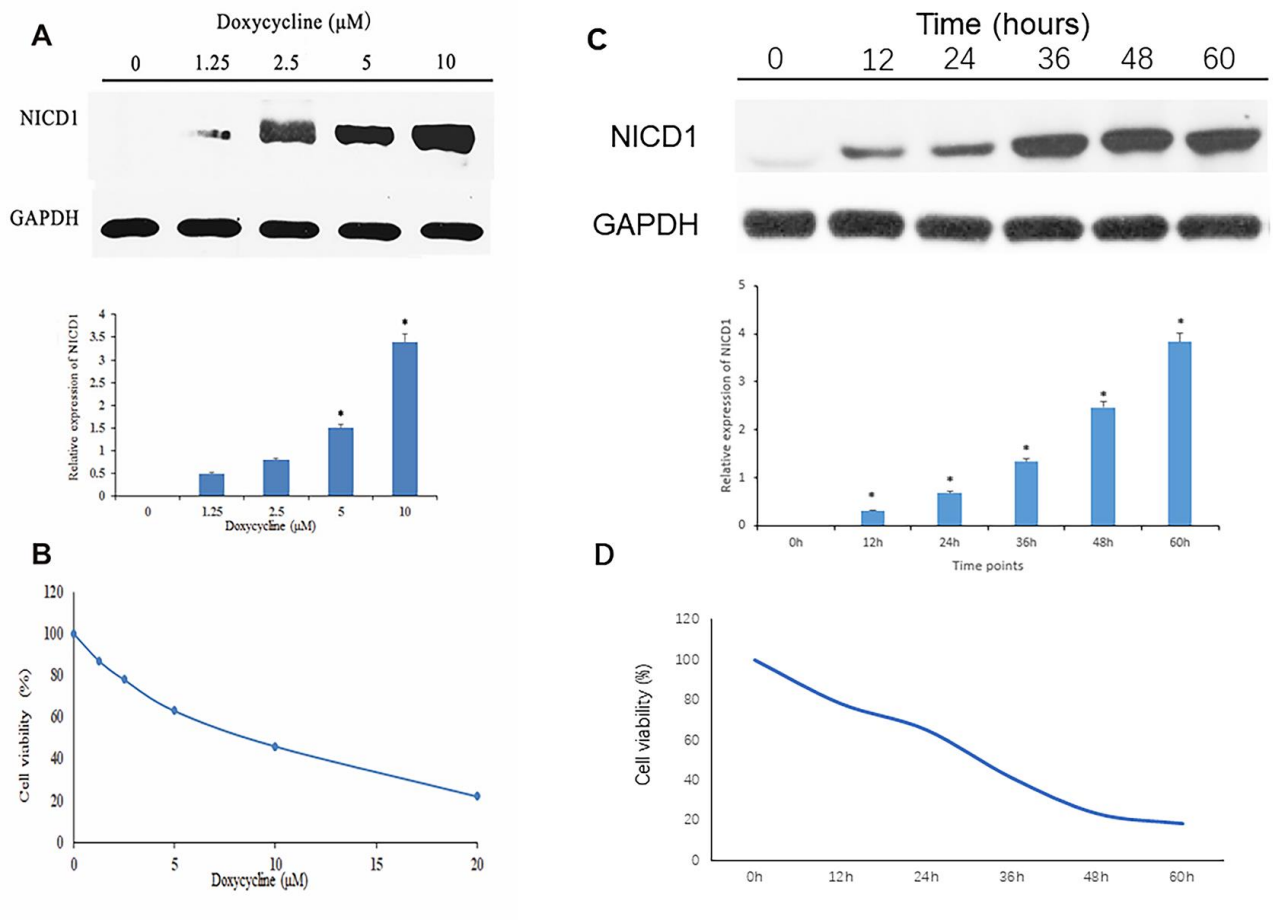
**Notch1 expression and cell viability induced by doxycycline treatment.** (**A**) The NICD1 expression induced by doxycycline treatment at various concentrations. GAPDH was used as loading control. *P<0.05, significantly different compared with the untreated control group. (**B**) Notch1 induced by doxycycline in various concentrations has inhibited cell viability in human osteosarcoma cell (MG-63). All the experiments were conducted in triplicates. (**C**) The NICD1 expression induced by doxycycline treatment at various time points including 0h, 12h, 24h, 48h, 36h and 60h. GAPDH was used as loading control. *P<0.05, significantly different compared with the untreated control group. (**D**) Notch1 induced by doxycycline in various time points including 0h, 12h, 24h, 48h, 36h and 60h has inhibited cell viability in human osteosarcoma cell (MG-63). All the experiments were conducted in triplicates.

To ensure that TT-NOTCH1 cells expressed functional Notch1 protein in response to doxycycline treatment, we carried out an experiment before proceeding to *in vitro* studies. As shown in Figure 1A, Notch1 protein (NICD1) expression of TT-NOTCH1 was significantly induced by doxycycline at 5 μM and 10 μM. This result was consistent with a previous observation [10]. To investigate the anti-proliferative effect of Notch1 induced by doxycycline on osteosarcoma cells, human osteosarcoma MG-63-Notch1 (TT-NOTCH1) cells were treated with doxycycline and analysed by MTT assays (Figure 1B). After treatment, the viability of osteosarcoma cells was significantly decreased in a dose-dependent manner, with an IC_50_ value of 7.60 μM. This result showed that Notch1 activated by doxycycline inhibited osteosarcoma cell proliferation in a dose-dependent manner.

### Notch1 induces cell cycle arrest at S phase

**Figure 2:**
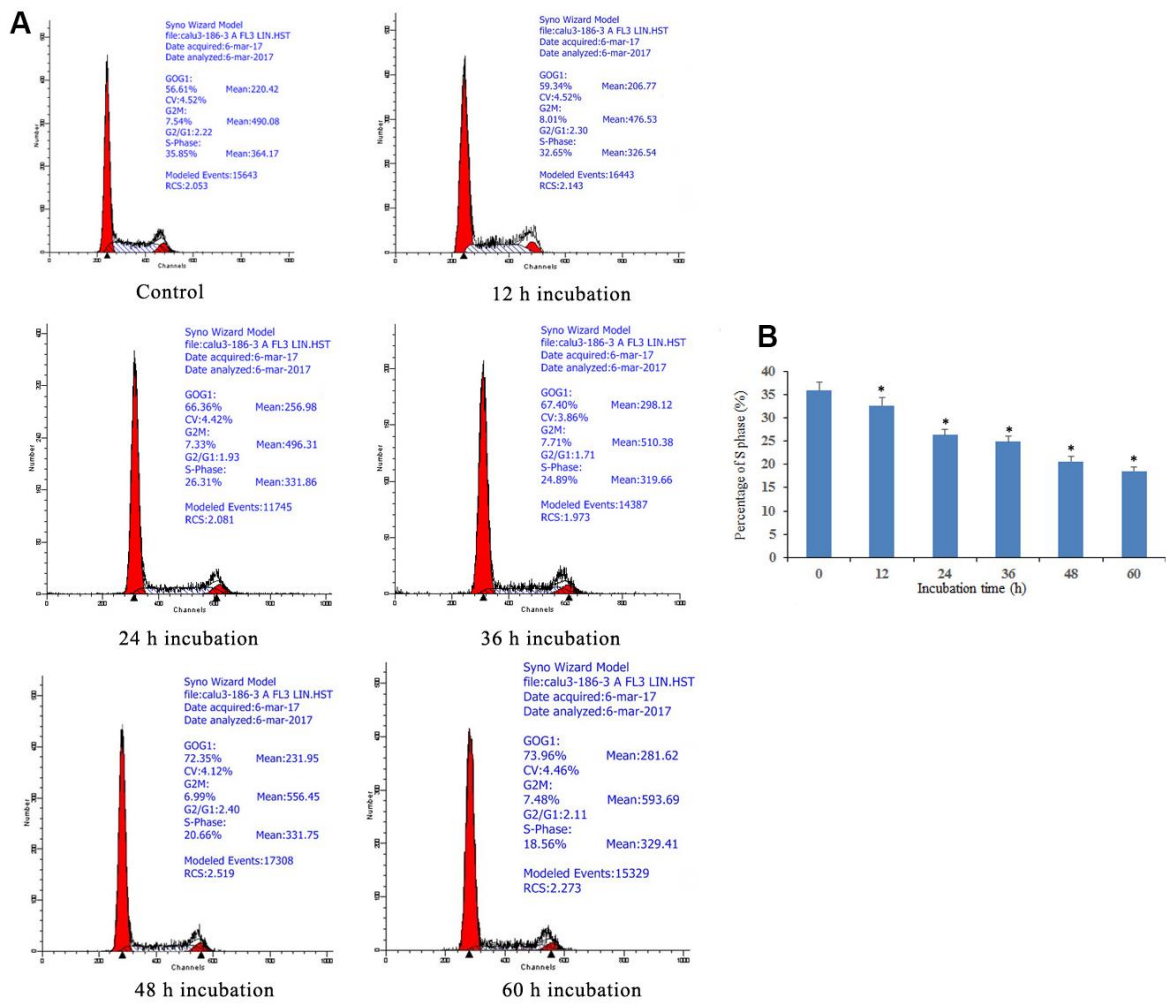
**Notch1 induced S phase cycle arrest in human osteosarcoma cells.** (**A**) Notch1 induced by doxycycline in different time induced S phase cell cycle arrest in human osteosarcoma cells. Cells were treated with doxycycline for 0, 12, 24, 36, 48 and 60 h and analyzed by flow cytometry. The percentage of cell population at G1, S, and G2/M phases were represented as mean ± SD of three independent experiments. (**B**) The histogram of percentage of S phase in different groups. *P<0.05, significantly different compared with the untreated control group. All the experiments were conducted in triplicates.

The cell cycle arrest via doxycycline-induced Notch1 is shown in Figure 2. After treatment with doxycycline at 10 μM for 12, 24, 36, 48, 60 h, based on previous experiments (data not shown), the percentages of S phase cells were 35.85%, 32.65%, 26.31%, 24.89%, 20.66% and 18.56%, respectively. These results show that doxycycline upregulates the expression of Notch1 over time, which inhibits the rate of cell proliferation by decreasing the percentage of S phase cells.

### Notch1 induces apoptosis of osteosarcoma cells

**Figure 3:**
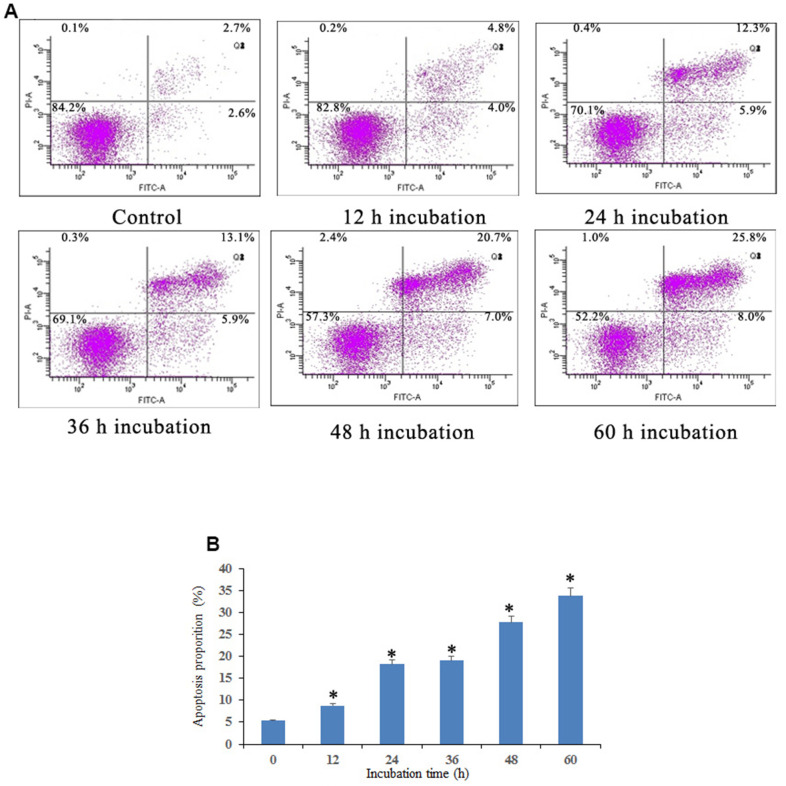
**Notch1 induced osteosarcoma cell apoptosis.** (**A**) Notch1 induced by doxycycline in different time induced osteosarcoma cell apoptosis. Cells were treated with doxycycline for 0, 12, 24, 36, 48 and 60 h and analyzed using Annexin V-FITC/PI flow cytometry. (**B**) The histogram indicated that apoptosis proportion from three separate experiments in different groups. *P<0.05, significantly different compared with the untreated control group. All the experiments were conducted in triplicates.

Apoptosis induced by Notch1 was further investigated through a Dead Cell Apoptosis Kit (Invitrogen) containing recombinant Annexin V conjugated to FITC and a ready-to-use solution of red-fluorescent propidium iodide (PI) nucleic acid binding dye. As shown in Figure 3, osteosarcoma cells were found to exhibit different degrees of cell shrinkage, chromatin condensation and nuclear fragmentation after treatment with 10 μM doxycycline for 12, 24, 36, 48, and 60 h. Annexin V-FITC/PI double staining demonstrated that osteosarcoma cells activated the Notch1 pathway in a time-dependent manner after treatment with doxycycline in both early and late apoptotic cells, in which the proportion of apoptotic cells increased from 5.3% to 33.8%.

**Figure 4:**
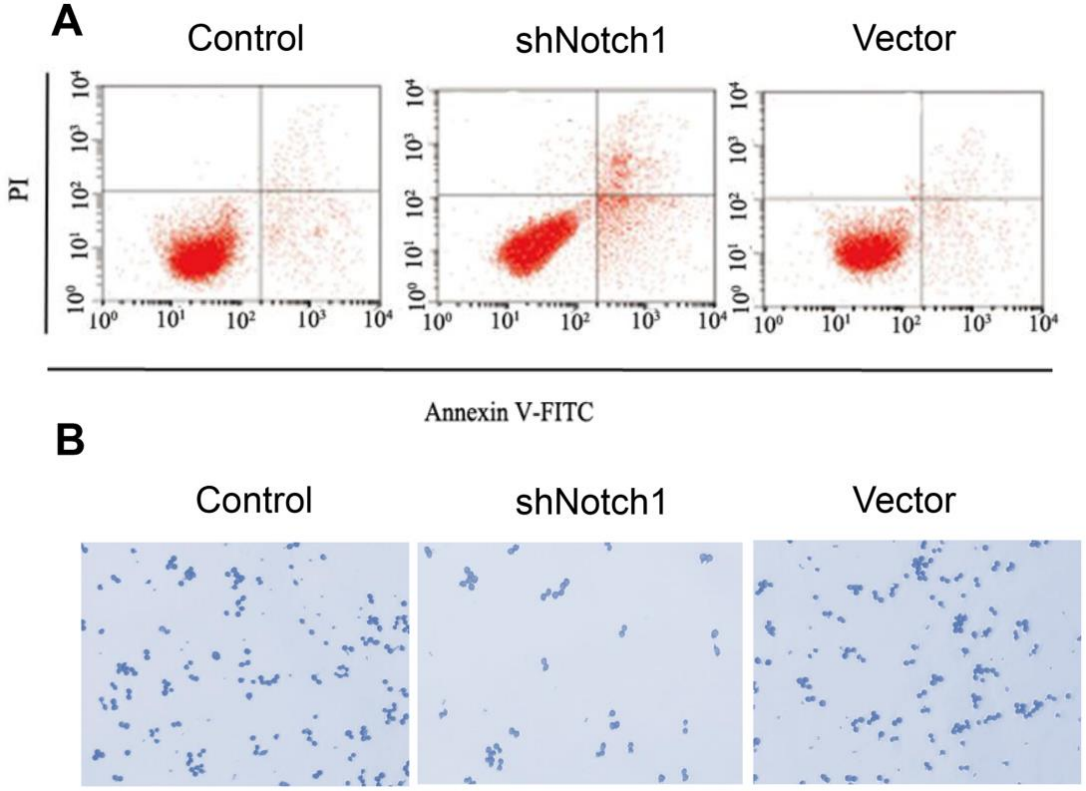
**shNotch1 induced osteosarcoma cell apoptosis and cell proliferation.** (**A**) shNotch1 treatment induced up-regulated osteosarcoma cell apoptosis. (**B**) shNotch1 treatment induced up-regulated osteosarcoma cell colony formation.

In advanced experiments, we analysed the effect of shNotch1 treatment on TT-NOTCH1 cells. It was found that shNotch1 could upregulate the apoptosis rate and improve cell colony formation (Figure 4A, 4B).

### Notch1 activates autophagy in osteosarcoma cells

**Figure 5:**
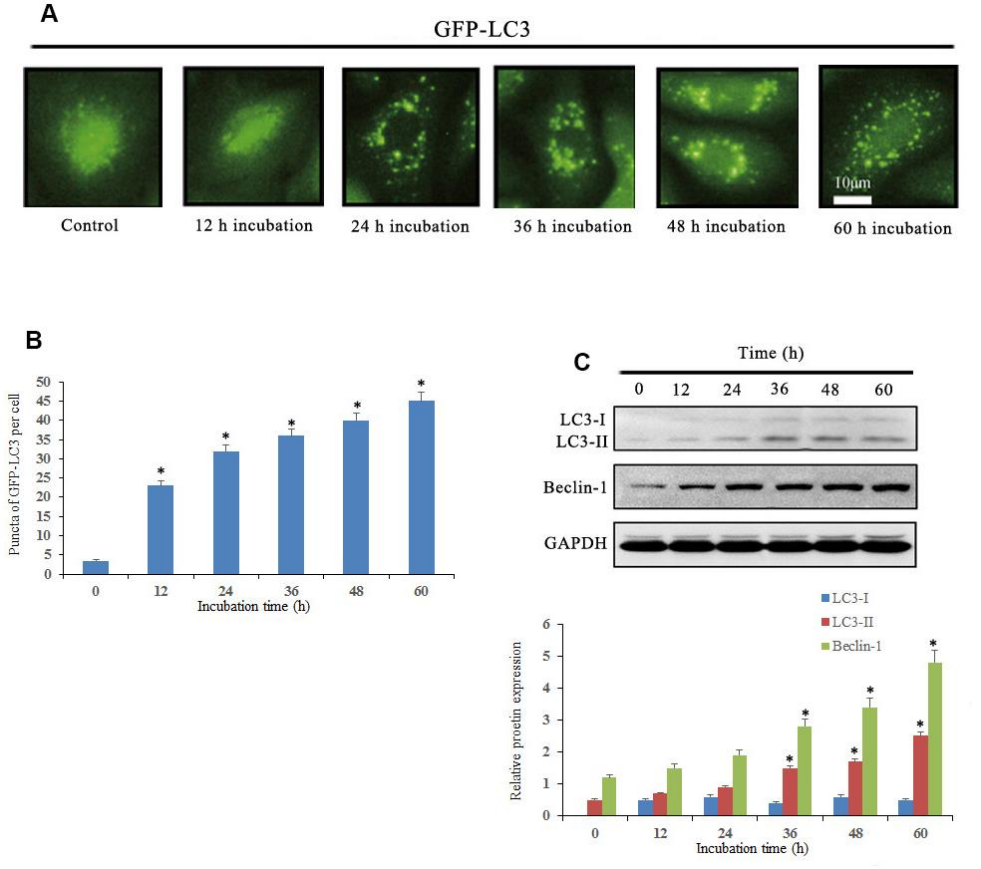
**Notch1 induced autophagy in human osteosarcoma cells.** (**A**) Osteosarcoma cells were transiently transfected with GFP-LC3 plasmid for 24 h and then treated with doxycycline (10 μM) for 0, 12, 24, 36, 48 and 60 h. Doxycycline treated cells displayed a punctate pattern of GFP-LC3 expression, which represented formation of autophagosomes. (**B**) The histogram indicated that autophagy proportion from three separate experiments in different groups. *P<0.05, significantly different compared with the untreated control group. (**C**) Autophagy-related proteins, LC3 and beclin-1, were analyzed by western blotting. *P<0.05, significantly different compared with the untreated control group. All the experiments were conducted in triplicates.

We then determined whether Notch1 induced by 10 μM doxycycline could induce autophagy in osteosarcoma cells. The results showed that the average number of GFP-LC3 puncta per cell was 4.6, 23.4, 32.2, 35, 37.2 and 42.2, which suggested that treatment of osteosarcoma cells with doxycycline resulted in a significant increase in GFP-LC3 puncta formation (Figure 5). Furthermore, we investigated the expression of autophagy-related proteins by Western blotting. The results showed that doxycycline-induced Notch1 increased the levels of LC3B-II and Beclin-1 in osteosarcoma cells in a time-dependent manner.

### Notch1 blocks the PI3K/Akt/mTOR signalling pathway

**Figure 6:**
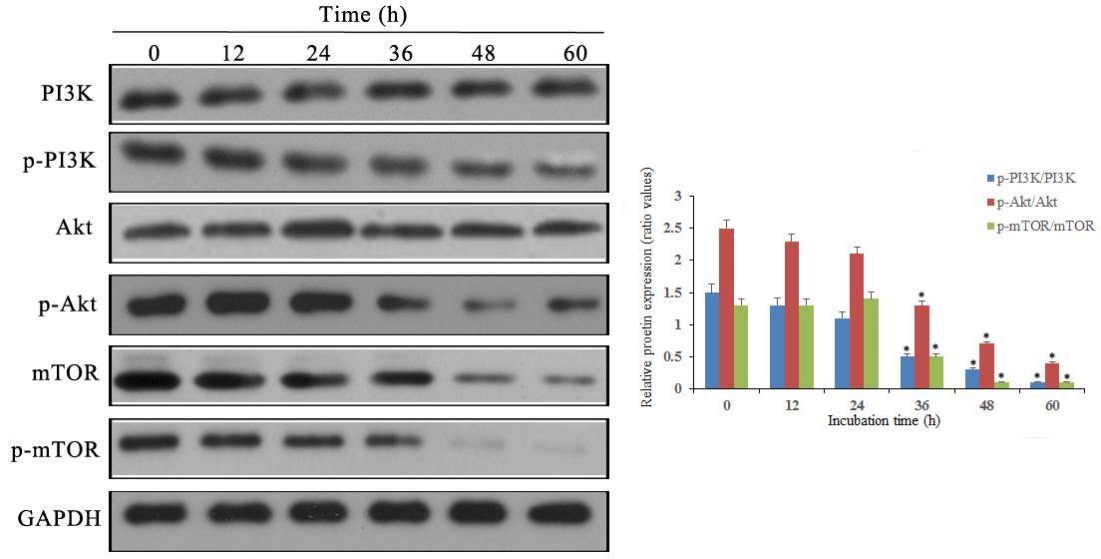
**Notch1 induced by doxycycline has blocked PI3K/Akt/mTOR signaling pathway in human osteosarcoma cells.** Levels of PI3K, p-PI3K, Akt, p-Akt, mTOR, p-mTOR were analyzed by western blotting. *P<0.05, significantly different compared with the untreated control group. All the experiments were conducted in triplicates.

**Figure 7:**
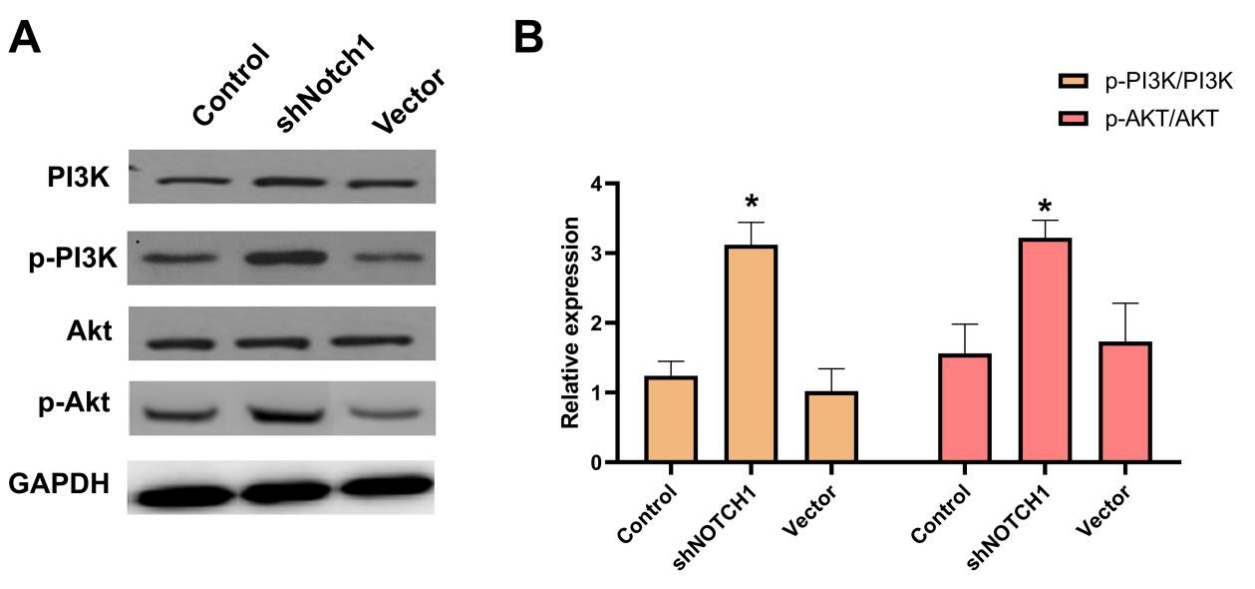
**shNotch1 up-regulated the PI3K/Akt signaling pathway in human osteosarcoma cells.** (**A**) The graphs of western blots were present. (**B**) Levels of PI3K, p-PI3K, Akt and p-Akt were analyzed by western blotting. *P<0.05, significantly different compared with the untreated control group. All the experiments were conducted in triplicates.

Western blot results showed that p-PI3K, p-Akt and p-mTOR were significantly downregulated after doxycycline treatment compared to those of the control group (Figure 6). These data suggest that doxycycline-induced Notch1 expression inhibits the PI3K/Akt/mTOR signalling pathway, which may be the molecular mechanism of apoptosis and autophagy. Compared with that of the control group, silencing Notch1 could lead to reduced expression of p-PI3K and p-Akt; however, the silencing effect was not detected in the vector-only group (Figure 7).

## DISCUSSION

Notch signalling plays important roles in determining cell fate during development and is activated upon cell-to-cell contact as a result of interactions between Notch receptors and their ligands [17]. Ligand binding results in proteolytic cleavage of Notch receptors by γ-secretases, leading to the release of an 80-kDa intracellular domain of Notch (NICD), which then translocates to the nucleus and activate the transcription of downstream target genes [18]. Our study showed that high expression of Notch1 positively correlated with active autophagy in human osteosarcoma cells. Upregulated Notch1 activated autophagy and cell death related to autophagy, which suggested the potential role of Notch1 in modulating the activity of autophagy.

To investigate the effect of Notch1 on osteosarcoma cell growth and autophagy, we developed an *in vitro* model of doxycycline-induced expression of NICD1 using the TET-ON system, which was based on the tetracycline-controlled transactivator protein tTA [19]. Our *in vitro* study showed that overexpression of NICD1 in osteosarcoma cells was significantly reduced by doxycycline treatment. Further results showed that overexpression of Notch1 could inhibit osteosarcoma cell proliferation and improve apoptosis and autophagy by regulating the PI3K/Akt/mTOR pathway.

The Notch pathway is critical for development and tissue homeostasis and consists of ligands, receptors, and intracellular signalling molecules. Notch signalling is abnormally activated in various cancers, such as leukaemia, and malignant solid tumours, such as lung, prostate, and breast cancers. Yao et al. reported that Notch1 activation induced cell cycle arrest and apoptosis in human cervical cancer cells [20]. Greenblatt et al. showed that activating Notch1 signalling induced apoptosis in medullary thyroid cancer cells [21]. Zeng et al. suggested that the Notch1 signalling pathway may inhibit A549 cell growth by regulating cell cycle-related and anti-apoptotic protein expression by regulating the PI3K/Akt pathway [22]. The present study proves that Notch1 induces osteosarcoma apoptosis.

Apart from apoptosis, autophagy also plays a significant role in determining cell fate [23]. The present study showed that the Notch1 pathway could induce autophagy by upregulating LC3-II and Beclin-1 expression. However, Yao et al. showed that the expression levels of LC3-II and Beclin-1 were significantly increased in Notch1 siRNA-transfected U251 cells, suggesting that cell autophagy was induced when Notch1 was downregulated in glioma cells [24]. These results were opposite to those of the present study. Thus, the relationship between the Notch1 pathway and cancer cell autophagy needs further confirmation.

Recent studies have shown that multiple signalling molecules are related to apoptosis and autophagy [25]. A close connection between autophagy and angiogenesis/apoptosis has also been demonstrated in osteosarcoma. A previous study revealed that tetrahydrocurcumin suppressed angiogenesis by targeting HIF-1α and autophagy in human osteosarcoma cells [26]. In addition, Li HY et al. reported that celastrol induced apoptosis and autophagy via the ROS/JNK signalling pathway in human osteosarcoma cells [27]. The PI3K/Akt/mTOR pathway is genetically targeted in many kinds of tumours, and it is frequently activated as a cancer driver. PI3K and Akt are vital mediators of carcinogenesis through phosphorylation. One major downstream effector of Akt signalling necessary for tumourigenesis is mTOR, which has also been reported to participate in the regulation of autophagy in mammalian cells [28]. Research has suggested that many monomers of traditional Chinese herbs play important roles in anticancer processes by inactivating the PI3K/Akt/mTOR pathway. For example, G2/M cell cycle arrest and apoptosis were induced by oridonin via the PI3K/Akt signalling pathway in hormone-independent prostate cancer cells [29]. Activation of PI3K/Akt signalling pathways antagonized sinomenine-induced lung cancer cell apoptosis [30].

Autophagy has a dual role in the regulation of cell death. Mild autophagy protects cells from harmful conditions, and severe or rapid autophagy induces programmed cell death, which is called autophagic cell death [31, 32]. In previous studies, cooperation between autophagy and apoptosis was identified and was suggested to promote cell death [33, 34]. In this study, our data showed that doxycycline significantly increased cell autophagy, which indicated a synergistic role of autophagy death and apoptosis after doxycycline treatment. A previous study reported that doxycycline suppresses cell proliferation and matrix metalloproteinase (MMPs) activity and induces apoptosis in human osteosarcoma cells *in vitro*. This anti-proliferative activity of doxycycline is attributed to the inhibition of MMPs, enzymes involved in the degradation and remodelling of the extracellular matrix. Herein, the ability of doxycycline to inhibit MMPs and induce autophagy can represent new potential targets for the treatment and detection of cancer patients.

The present study showed that Notch1 inhibited the phosphorylation of PI3K, Akt and mTOR. Thus, PI3K/Akt/mTOR signalling is central to the Notch1 signalling pathway, suggesting that targeting PI3K/Akt/mTOR with small molecule inhibitors would show great promise in treating osteosarcoma. Better understanding of how Notch1 regulates PI3K/Akt/mTOR would likely create novel therapeutic opportunities for targeting these important cellular pathways in osteosarcoma.

## CONCLUSIONS

In conclusion, our study showed that Notch1 activation could suppress cell proliferation by causing S phase arrest and induce apoptosis and autophagy in human osteosarcoma cells. In addition, Notch1 induced apoptosis and autophagy through the inhibition of the PI3K/Akt/mTOR signalling pathway, which suggested that Notch1-induced autophagy was a pro-survival process. Taken together, our findings provide an alternative strategy of combining regulators of Notch1 expression to treat osteosarcoma.

## MATERIALS AND METHODS

### Cell lines

The Notch1-expressing MG-63 cell line, TT-NOTCH1, was established as previously described [10]. Conditional expression of Notch1 was achieved using the TET-ON system. TT-NOTCH1 cells were cultured in DMEM (Thermo Fisher Scientific, MA, USA) supplemented with 5% FBS (Invitrogen, CA, USA), 100 U/mL penicillin and 100 pg/mL streptomycin (Sigma, MO, USA) at 37° C in a humidified incubator supplemented with 5% CO_2_ and 95% air.

### shNOTCH1 construction

The plasmid used in this experiment was purchased from Shanghai Jikai Gene Chemical Technology Co., Ltd. The shRNA that silences Notch1 is called shNotch1, and the negative control group is shNotch1-NC. Inoculate MG-63 cells in a 6-well plate in advance, follow the transfection instructions to find the most suitable conditions for transfection. After transfection for 48h, the inhibition efficiency of Notch1 was detected by western blot. The sequences of shNotch1 and control vector were as follows: shNOTCH1: 5’-AAGACATGACCAGTGGCTA-3’ and the control vector: 5’-TTCTCCGAACGTGTCACGT-3’.

### MTT assay

Cells were subcultured in a 96-well plate at a density of 2×10^4^ cells/mL. Various concentrations of doxycycline (1.25, 2.5, 5, 10 and 20 μM) were added, and the plate was incubated for 24 h. Cell viability was examined using the 3-(4,5-dimethylthiazol-2-yl)-2,5-di-phenyltetrazolium bromide (MTT) assay. Then, 20 μL of MTT solution (5 mg/mL) was added into each well and incubated for 4 h. The absorbance at 490 nm was measured after 150 μL of dimethylsulfoxide was added and shaken for 10 min. The cell proliferation curve was then drawn, and the proliferation efficiency was examined. The experiments were repeated three times independently.

### Cell cycle analysis

Cells from different groups were inoculated into 6-well plates at a density of 5×10^5^ cells/mL and treated for different incubation times (0, 12, 24, 36, 48 and 60 h) with 10 μM doxycycline (DOX) after cell adherence. The cells were collected, centrifuged and washed with PBS, and 70% ice-cold ethanol was added and incubated overnight. After centrifugation, 150 μL of propidium iodide and 150 μL of RNaseA solution were added for staining for 30 min at 4° C in the dark. BD FACSort Cell Quest software for flow cytometry was used to analyse the cell cycle and obtain cell cycle percentages.

### Apoptosis analysis

Cells were seeded in 6-well plates at a density of 5×10^5^ cells/mL and treated with 10 μM DOX. After incubation, the cells were collected at 0, 12, 24, 36, 48 and 60 h and washed with ice-cold PBS. Then, binding buffer containing Annexin V-FITC and PI was added to the cells. After being incubated for 15 min at room temperature in the dark, the samples were analysed by a flow cytometer (BD Biosciences).

### Autophagy analysis

Cells were seeded into 6-well plates at a density of 5×10^5^ cells/mL and treated with 10 μM DOX for 48 h. Then, the cells were incubated with monodansylcadaverine (MDC) for 15 min and immediately analysed under a fluorescence microscope (Leica Microsystems, Wetzlar, Germany).

### Western blotting

The cells were harvested and lysed on ice for 30 min in buffer. After centrifugation, the protein concentrations were determined, and the proteins were separated by 10% sodium dodecyl sulfate-polyacrylamide gel electrophoresis. Then, the proteins were transferred to a polyvinylidene fluoride (PVDF) membrane. After being blocked for 1 h, the membranes were incubated with specific primary antibodies overnight at 4° C followed by secondary antibodies for 2 h at room temperature. Then, the membranes were treated with chemiluminescence reagents (Santa Cruz) per the manufacturer’s instructions and analysed by Image J software.

### Statistical analysis

All statistical analyses were carried out using SPSS 17.0 software (SPSS, IL, USA). All the experiments were conducted in triplicates. The data were presented as the means ± standard deviation. Differences between two groups were analysed using Student’s t-test or chi-square test analysis. One-way ANOVA followed by Kruskal-Wallis H test was used for multiple comparisons. The results were considered significant when *P* value < 0.05.

### Availability of data and materials

The datasets used and/or analyzed during the current study are available from the corresponding author on reasonable request.

### Ethics approval and consent to participate

The present study was approved by the Institutional Animal Care and Use Committee of The Third Xiangya Hospital Central South University.
